# Importance of the Conservation of the First Permanent Molar

**Published:** 1902-11-15

**Authors:** A. C. Runyan


### IMPORTANCE OF THE CONSERVATION OF THE FIRST PERMANENT MOLAR.

BY A. C. RUNYAN, D.D.S.

Read before the Southwestern Michigan Dental Society, September 10, 1902.

I believe I fully appreciate the delicacy of the subject, as the opinions that are held regarding the first permanent molar are almost as numerous as there are individual dentists. This, however, only proves the importance of this particular tooth to the dental practitioner.

To me it has been one of my greatest stumbling blocks, and I believe I have put more thought on this particular tooth and its importance to the dental arch, than to any one branch of my practice.

Having had over twenty years experience in one locality. I have had opportunities to observe the changes that may be brought about by the extraction of the first molar at an improper time, and I will endeavor to show by models and some crude drawings, what has been the result of my observations. I have only had time to prepare models of some of the cases that have come into my office since the subject was assigned to me about three weeks since, and I hope that at some future meeting this subject may be brought up again, and that the few points that I may bring out here may stimulate others to make observations along these lines and when opportunities present themselves to take impressions of the deformities that have been caused by the extraction of the first permanent molars, and I believe there will be an extra effort made to bring about some way in which work may be done along the proper lines for the conservations of these important teeth.

I remember reading an article published in one of our journals some years since, recommending the radical removal of the first molars, contending that they came in so early that they were only intended as a sort of make-shift till it was time for the second and third molars to erupt. Another argument advanced was that by removing them the other molars could erupt properly without being crowded.

I can show by a case of which I have the models what are the results following such advice, where the lower first molars were extracted too soon. In this case the lower first molars were extracted just before or at about the time that the temporary molars were lost. Mark the result. The anterior six upper occlude far outside of the lower. The removel of the first molars and the natural loss of the temporary molars left nothing for the back teeth to rest on, the result is the lower anterior teeth were permitted to bite against the upper gum and when the bicuspids erupted they were not permitted to assume their natural length, and the occlusion is permanently spoiled. There is enough in one such case for any ortho-dental specialist to write several papers on.

Another case, the models illustrate the effect produced when the first upper right molar was extracted too soon. There being nothing for the lower teeth to rest on, the jaw has been protruded forward and the upper anterior teeth have dropped back, till there is a permanent deformity which could not be remedied by the extraction of the lower molar later on, and the occlusion was such that it has an inclination to increase rather than diminish the difficulty.

Many of you may think that these are extreme cases, but if the average practitioner will bear this subject in mind and by careful observation he will be surprised at the number of cases that will come to his notice, and he will find that these are just average cases.

Another model shows the loss of the first lower molars, and the second upper bicuspids, after the second molars have been erupted, and the pre-molars have assumed their normal position in the arch. Mark the difference in the results. There has been but little serious injury done either to the proper occlusion of the teeth or to the shape of the jaw. I am not going to tell you how to avoid these results but will say that from my observation and the models I have collected, it is very necessary that the permanent molars be kept in place till the second are thoroughly erupted, or the bicuspids are in position to perform their proper functions.

DISCUSSION.

Dr. Cigrand: We are extracting too many first permanent molars. This makes work only for the prosthetic dentist, I have taken special care to save the first molars in my own child's mouth because of the value I attach to this tooth. It is often difficult to save this tooth, and it may be that our efforts should be directed to informing parents of the importance of early attention to this tooth. They very often believe it is one of the deciduous teeth and hence do not give it the care they would if informed of its nature and value. Time so spent is well utilized and deserving of as good a fee as for any other service. When a child presents himself for extraction of such a tooth a few minutes time will convince him that the tooth should be saved, and he will convince his parents of the fact perhaps better than the dentist could do. Convince the child that when decay attacks a tooth it does not necessarily imply that it is doomed.

My own daughter has a very frail lower molar that I am carefully keeping by cement fillings until such time as I may be able to crown it with a gold cap crown. When I hope to stop the process of disintegration and make of it a useful tooth.

An eminent authority on diseases of the alimentary canal says that three-fourths of the cases of indigestion result from diseased teeth or impaired dental structures, of one kind or another. Dentists who neglect the first molars, or extract them without the best of reason are in my judgment guilty of malpractice. When these teeth are extracted a plate or bridge should replace them in order that the normal developement of the jaws will take place.

Dr. Warboys: The trouble is that patients seek us not for advice but for an operation. We would not think much of a surgeon who would amputate a finger because of a felon or other injury which might be cured. If we should take a position in the matter of extracting these teeth, our patients would soon come to think more of their teeth and us too because of our advice.

Dr. Runyan: I have studied this matter for a long time and have also noted the results of the extracting of these teeth and this has convinced me that it is a bad practice. Formerly every one thought it was the only thing to do to remove these teeth, but the sentiment seems to be changing to a more conservative practice.

Dr. Taft: The best condition of the first permanent molar implies the early care of the deciduous teeth. Not only should ordinary cleanliness of the child's teeth be impressed upon the minds of parents, but they should also be made more intelligent in regard to such foods as may be helpful in building up the permanent teeth, various sicknesses to which children are subjected should also be cared for in regard to the future or developing teeth. The loss of the first permanent molar not only implies the loss of one tooth, but it means a defective function on that side of the mouth. Perfect nutrition is not practicable with the first part of this function badly defective.

Dr. Runyan: The time during which the first molar is in process of building and even up to its eruption is a time in the child's life when it is liable to many diseases which would interfere especially with a perfect developement of its crown. As is evidenced by frequent appearance of abnormal lines, pits, etc. For this reason they are more susceptible to decay producing influence.

